# In vitro and in vivo activity of R- and S- praziquantel enantiomers and the main human metabolite trans-4-hydroxy-praziquantel against *Schistosoma haematobium*

**DOI:** 10.1186/s13071-017-2293-3

**Published:** 2017-08-01

**Authors:** Jana Kovač, Mireille Vargas, Jennifer Keiser

**Affiliations:** 1 Department of Medical Parasitology and Infection Biology, Swiss Tropical and Public Health Institute, Basel, Switzerland; 2 University of Basel, Basel, Switzerland

**Keywords:** Praziquantel, *Schistosoma haematobium*, In vivo, In vitro

## Abstract

**Background:**

Praziquantel (PZQ) is the mainstay of schistosomiasis control and has been successfully used for decades. However, its mechanism of action is not fully understood. While the majority of studies have been conducted on *Schistosoma mansoni*, it is not known which enantiomer, R- or S-praziquantel (R-/S-PZQ), is responsible for the activity on *Schistosoma haematobium*.

**Methods:**

In vitro and in vivo studies were conducted to evaluate the activity of R- and S-PZQ, racemic PZQ and the main human metabolite, namely *trans*-4-OH-PZQ, on *S. haematobium*. IC_50_ values on adult *S. haematobium* were determined in vitro. Dose-response relationship studies were performed in golden Syrian hamsters, harbouring a chronic *S. haematobium* infection.

**Results:**

R-PZQ displayed the highest activity against adult worms in vitro, revealing an IC_50_ of 0.007 μg/ml at 4 h and 0.01 μg/ml at 72 h. In contrast, S-PZQ was 501× less active (eudysmic ratio at 4 h), with an IC_50_ of 3.51 and 3.40 μg/ml (4 and 72 h, respectively). Racemic PZQ and *trans*-4-OH-PZQ resulted in an IC_50_ of 0.03 μg/ml and 1.47 μg/ml both at 4 and 72 h, respectively. In vivo, R-PZQ was the most potent drug with worm burden reductions (WBRs) of 98.5, 75.6 and 73.3% at 125.0, 62.5 and 31.0 mg/kg, respectively. A single oral dose of 250.0 mg/kg PZQ resulted in a WBR of 99.3%. S-PZQ was highly active in vivo at 250.0 and 500.0 mg/kg with WBRs of 83.0 and 94.1%, respectively. The lowest tested dose of S-PZQ, 125.0 mg/kg, showed moderate activity (WBR of 46.7%). The calculated ED_50_ for R- and S-PZQ were 24.7 and 127.6 mg/kg, respectively, with a corresponding eudysmic ratio of 5.17.

**Conclusion:**

Our data support the theory of R-PZQ driving the antischistosomal activity. Interestingly, also S-PZQ proved to possess a significant activity towards *S. haematobium*, particularly in vivo.

## Background

Schistosomiasis is one of the prominent neglected tropical diseases (NTDs), caused by blood-dwelling flukes of the genus *Schistosoma*. It is affecting over 230 millions of people around the world, mostly concentrated in poor, tropical and subtropical areas [1–3].

Intestinal schistosomiasis, caused by *S. mansoni* and *S. japonicum*, manifests with abdominal symptoms (e.g. blood in stool, abdominal discomfort, diarrhoea) and can lead to liver failure [4, 5], while *S. haematobium* causes urinary schistosomiasis, triggering bladder pathology, often resulting in bladder cancer [4, 6]. In addition, schistosomiasis influences the course and outcome of pregnancy and affects child’s intellectual and physiological development [7, 8].

Praziquantel (PZQ) is the only effective drug available against schistosomiasis and has been successfully in use for decades [4, 6, 9–13]. Originating from veterinary medicine and repurposed for human use, it has been thoroughly studied; however, knowledge regarding the mechanism of action is scarce [9, 11]. PZQ is a racemic compound consisting of two enantiomers, R- and S-PZQ [9, 11]. While there have been some in vitro and in vivo studies on the activity of PZQ, they mostly studied the racemic drug [14–20]. In the few studies, which explored the activity of either R- or S-PZQ, the reported findings vary. Nevertheless, most studies reported greater activity of R-PZQ over S-PZQ [21–24]. Staudt et al. [24] suggested that the main metabolite, R-*trans*-4-hydroxy-praziquantel (*tran*s-4-OH-PZQ), also possesses a high antischistosomal activity on *S. mansoni*. A similar finding was reported by Xiao et al. [25] for *S. japonicum*.

It is worth highlighting that the above-mentioned studies, testing the enantiomeric activity of R- and S-PZQ, were conducted using exclusively *S. mansoni* and *S. japonicum*. *Schistosoma haematobium* remains largely unexplored, regardless of the fact that it is responsible for the largest number of infections [26]. One of the many reasons of negligence might be the life-cycle of the parasite, which is difficult to maintain in laboratory conditions [27–29]. However, drug activity should be carefully elucidated on *S. haematobium* as well, since there is evidence that the activity of drugs, e.g. PZQ, oxamniquine or metrifonate, differs between species of the parasite [18, 30].

In this study, the activity of both PZQ enantiomers, R- and S-PZQ, as well as the racemic drug and the main metabolite (*trans*-4-OH-PZQ) was assessed on *S. haematobium*. The activity of all entities was tested in vitro on adult worms and the results were reported as IC_50_ values. The *S. haematobium* hamster model was used for testing different dosages of R-PZQ and S-PZQ compared with racemic PZQ in vivo. ED_50_ values were reported and worm burden reductions (WBRs) were compared between different treatment groups of R- and S-PZQ and the control group.

## Methods

### Drugs, media and animals

Pure analytes, R-, S- and *trans*-4-OH-PZQ were kindly supplied by Merck (Darmstadt, Germany). Racemic PZQ was purchased from Sigma Aldrich (Buchs, Switzerland). Drugs for in vitro studies were dissolved in dimethyl sulfoxide (DMSO; Fluka, Buchs, Switzerland). A mixture of 7% (vol/vol) Tween 80 and 3% ethanol (vol/vol) was used to suspend the drugs for in vivo treatment.

For cultivating adult schistosomes, standard RPMI 1640 medium (Life Technologies, Carlsbad, CA, USA) with addition of 5% heat-inactivated foetal calf serum (iFCS), 100 U/ml of penicillin (Life Technologies) and 100 μg/ml of streptomycin (Life Technologies) was used.

Thirty LVG golden Syrian hamsters (male, weight approximately 150 g), infected with approximately 350 cercariae of *S. haematobium* each, were obtained from the biomedical research institute (NR-21966, Rockville, MD, USA). The animals were kept under controlled conditions (22 °C, 50% humidity, 12/24 h of light and free access to water and rodent diet) to allow development of chronic infection 3 months post-exposure.

### In vitro and in vivo studies

#### In vitro studies

Adult worms were tested at a range of 0.01–3.00 μg/ml for R-PZQ and PZQ, at 0.1–30.0 μg/ml for S-PZQ and 0.1–3.0 μg/ml for *trans*-4-OH-PZQ. Drugs were prepared in medium using serial dilutions in flat bottom 24-well plates (BD, Falcon, Corning, NY, USA). Control wells consisted of 0.3% DMSO, which was the highest concentration of DMSO used to dissolve the drugs. Three months post-infection, *S. haematobium*-infected hamsters were euthanized with CO_2_ and dissected. Adult worms were collected from hepatic portal and mesenteric veins. Two to three worms, sexes equally represented, were placed per well and each concentration of the drug was tested in duplicates. Worms were incubated at 37 °C and 5% CO_2_ and the phenotypic changes were evaluated 1, 4, 24, 48 and 72 h post-incubation using a motility scale ranging from 3 (normal activity) to 0 (no activity, granularity present).

IC_50_ values were calculated with CompuSyn® software (version 1.0) from motility values at different concentrations of each drug. The linear correlation coefficient (r value) reflects the conformity of the experimental data. The eudysmic ratio was calculated using the following formula: IC_50_ distomer/IC_50_ eutomer, where R-PZQ is the eutomer and S-PZQ the distomer.

#### In vivo studies

Infected *S. haematobium* hamsters in groups of 3–4 were treated 3 months post-infection with a single oral dose of 250.0 mg/kg PZQ, 125.0 mg/kg R-PZQ, 62.5 mg/kg R-PZQ, 31.0 mg/kg R-PZQ, 500.0 mg/kg S-PZQ, 250.0 mg/kg S-PZQ or 125.0 mg/kg S-PZQ. 10 days post-treatment hamsters were euthanized with CO_2_ and dissected. Adult worms from intestinal veins were counted and sexed and the liver was inspected for live/dead worms and eggs. The control group (untreated) was dissected at the same time and the mean worm burden of treated hamsters was compared with untreated hamsters to determine the WBR. ED_50_ and eudysmic ratios were calculated as described above.

### Statistics

Statistical tests were performed using Prism software (version 7.03, GraphPad, CA, USA). Unpaired t-test allowing for unequal variances was used to determine differences in worm burden between the control group and the treatment groups. *P* < 0.05 was considered to be significant.

## Results

### In vitro studies

In vitro IC_50_ and IC_90_ values (4 and 72 h of incubation) of racemic PZQ, pure enantiomers and *trans*-4-OH-PZQ obtained against adult worms of *S. haematobium* are summarised in Table 1. The IC_50_ of R-PZQ was 0.007 μg/ml at 4 h and 0.01 μg/ml at 72 h, while S-PZQ was 501× less active (eudysmic ratio at 4 h) yielding IC_50_ values of 3.51 and 3.40 μg/ml (4 and 72 h, respectively). The IC_50_ of PZQ was 0.03 μg/ml, which is 4.3× higher compared to R-PZQ. *Trans*-4-OH-PZQ revealed an IC_50_ of 1.47 μg/ml at 4 and 72 h, respectively.

**Table 1:** IC_50_ and IC_90_ values of PZQ, R-PZQ, S-PZQ enantiomers and *trans*-4-OH-PZQ against adult worms of *S. haematobium*

	IC_50_ at 4 h (μg/ml)	r-value	IC_50_ at 72 h (μg/ml)	r-value	IC_90_ at 72 h (μg/ml)	r-value	Eudysmic ratio
PZQ	0.03	0.978	0.03	0.965	0.09	0.978	501
R-PZQ	0.007	0.803	0.01	0.940	0.03	0.940	
S-PZQ	3.51	0.925	3.40	0.923	5.98	0.923	
*Trans*-4-OH-PZQ	1.47	0.891	1.47	0.891	3.31	0.891	

### In vivo studies

Total WBRs and female WBRs following different single oral doses of R-, S-PZQ and PZQ are presented in Table 2. For all drugs and dosages tested, a higher activity on the female worms was observed. PZQ reduced the total worm burden by 99.3% at a single dose of 250.0 mg/kg. R-PZQ showed the highest total WBR at 125.0 mg/kg (98.5%) while with a half of the dose (62.5 mg/kg) the WBR was lower (75.6%). The lowest dose of R-PZQ, 31.0 mg/kg, yielded still a high total WBR of 73.3%. S-PZQ revealed a high activity at 500.0 and 250.0 mg/kg with total WBRs of 94.1 and 83.0%. A moderate total WBR of 46.7% was observed when the hamsters were treated with 125.0 mg/kg of S-PZQ. The calculated ED_50_s for R- and S-PZQ were 24.7 and 127.6 mg/kg, respectively, with a corresponding eudysmic ratio of 5.17. All female and total WBRs of the different treatment groups were significantly different from the control group (*P* < 0.05), except for the total WBRs of lowest doses of R- and S-PZQ (31.0 mg/kg and 125.0 mg/kg, respectively), which showed not to be significantly better compared to the control group.

**Table 2:** Worm burden reductions (WBRs) following different single oral doses of R, S-PZQ and PZQ

	No. of hamsters cured/treated	Mean no. of alive worms ± SD				WBR (%)	Female WBR (%)	ED_50_ (mg/kg)	
		Liver	Mesenteric veins	Total	Females				
Control	0/4	2.75 ± 3.8	31.0 ± 9.5	33.8 ± 16.8	15.3 ± 5.2	–	–	–	
PZQ									
250.0 mg/kg	3/4	0.25 ± 0.5	0	0.3 ± 0.5	0	99.3	100		
200.0 mg/kg^b^						77.2		118.1	
150.0 mg/kg^b^						66.1			
100.0 mg/kg^b^						39.2			
R-PZQ									
125.0 mg/kg	2/4	0.25 ± 0.5	0.25 ± 0.5	0.5 ± 0.6	0	98.5	100	24.7^a^	
62.5 mg/kg	1/4	5.0 ± 4.7	3.25 ± 3.6	8.3 ± 8.1	2.3 ± 3.3	75.6	85.2		
31.0 mg/kg	0/3	6.7 ± 11.5	2.3 ± 2.3	9.0 ± 13.9	4.0 ± 6.9	73.3	73.8		
S-PZQ									
500.0 mg/kg	3/4	1.25 ± 2.5	0.75 ± 1.5	2.0 ± 4.0	0	94.1	100	127.6^a^	5.17^c^
250.0 mg/kg	2/4	4.0 ± 4.9	1.75 ± 2.1	5.8 ± 6.9	0	83.0	100		
125.0 mg/kg	0/3	7.0 ± 6.2	11.0 ± 6.0	18.0 ± 12.1	0.7 ± 0.6	46.7	95.6		

^a^Determined on total worm burden reductions

^b^Data reported by Webbe & James [18]

^c^Eudysmic ratio

## Discussion

With no available alternative drug, PZQ is the mainstay of schistosomiasis control [9–12]. Apart from reliance on a single drug, an additional drawback is the large dose required, resulting in a huge size of the tablet, containing a racemic mixture of PZQ [31–33]. The discussion about the activity of each enantiomer of the drug, namely R- and S-PZQ, has been on-going and therefore it is time to conclude which enantiomer is responsible for the antischistosomal activity [9, 32–34]. Moreover, development of a paediatric PZQ formulation is currently ongoing and thorough examination of in vitro and in vivo activity of PZQ and its enantiomers will not only contribute to a better understanding of the drug but also aid to select the optimal entity for the final formulation (R-PZQ or racemic PZQ) [35]. While *S. mansoni* has been thoroughly researched, *S. haematobium* remains neglected in the laboratory, despite of being responsible for a large share of the burden of schistosomiasis [26, 29]. This holds true also for drug sensitivity testing including studies on PZQ. While a few studies assesed the activity of PZQ on *S. haematobium*, all of them only evaluated the activity of racemic PZQ [18, 19, 29]. Studies on *S. haematobium* are pivotal as many antischistosomals, oxamniquine, metrifonate and PZQ have very distinct profiles on the different schistosome species [30]. To our knowledge, our study is the first to investigate the activity of both enantiomers of PZQ, at three different doses, compared to a single dose of PZQ, in vivo. Additionally, the activity of both enantiomers was compared also to the main human metabolite, *trans*-4-OH-PZQ, and the racemic drug, in vitro. Our results show that R-PZQ is driving the antischistosomal activity of PZQ, both in vitro and in vivo. The IC_50_ value of racemic PZQ was 4.3× higher compared to the enantiopure R-PZQ in vitro*.* In vivo results followed a similar pattern: R-PZQ at 125 mg/kg resulted in WBRs above 98%, as did a twice higher dose of PZQ, 250 mg/kg. The latter result is in the line with findings from the dose-response relationship study with PZQ in *S. haematobium* infected hamsters conducted by Webbe & James, yielding an ED_50_ of 118 mg/kg [18].

Strikingly, it seems that in case of *S. haematobium* in contrast to *S. mansoni* [36], also S-PZQ possesses non-negligible activity. An ED_50_ of 127.6 mg/kg was calculated for S-PZQ, which is close to the value of the racemic drug. For comparison, *S. mansoni*-infected mice treated with 800 mg/kg S-PZQ showed only a low WBR of 19.6%. Hence, the eudysmic ratio is 64-fold lower for *S. haematobium* compared to *S. mansoni* [36]. However, it is worth highlighting that differences in the drug sensitivity between the two species might also be due to differences in the model, the hamster versus mouse model [27]. Finally, also *trans*-4-OH-PZQ revealed a 2.3 and 2.4-fold higher activity (72 and 4 h, respectively) against *S. haematobium* in vitro when compared to *S. mansoni*. A contribution of S-PZQ and *trans*-4-OH-PZQ to PZQ’s activity could explain the higher sensitivity to PZQ of *S. haematobium* when compared to *S. mansoni* in humans [30]. In humans, *S. haematobium* are residing in the venus plexus of the bladder, getting exposed mostly to high concentrations of S-PZQ and the metabolite, as a consequence of first pass metabolism. This is in contrast to *S. mansoni*, where the adult worms are exposed to un-metabolised drug in the mesenteric veins, prior to reaching the liver.

In addition, we observed increased sensitivity of female worms compared to the males in vivo for all entities studies. For example, the lowest dose of S-PZQ achieved a female WBR of 95.6%, while the males were only mildly affected (total WBR of 46.7%). The higher activity of PZQ on female worms has been reported previously [20].

As mentioned above, due to the difficulties maintaining the *S. haematobium* life-cycle, our in vivo data are based on a single experiment and the in vitro data on duplicate experiments. While we feel our data are robust and in line with standard procedures, in order to draw a final conclusion, which PZQ enantiomer to recommend for the therapy of *S. haematobium* infections, additional experiments would be beneficial.

## Conclusion

To conclude, we observed that R-PZQ possesses the highest activity among the PZQ enantiomers and main human metabolite tested against *S. haematobium.* Surprisingly, S-PZQ- showed a high activity in vivo. Additionally, the main human metabolite displayed an activity higher than S-PZQ in vitro. In the line with the current efforts to develop a paediatric formulation, an enantioselective R-PZQ formulation might bear some risk; however, further laboratory studies as well as clinical trials, including pharmacokinetic/pharmacodynamics relationship studies, would be required to confirm our findings.

## Abbreviations

ED_50_: Dose of the drug needed to reduce the worm burden by 50%
IC_50_: Concentration of the drug, needed to kill 50% of the parasites
PZQ: Praziquantel
WBR: Worm burden reduction
